# The stem of *Schisandra chinensis* and Schisandrin B alleviated DNCB-induced atopic dermatitis in mice by inhibiting the NF-κB pathway

**DOI:** 10.3389/fimmu.2026.1725312

**Published:** 2026-01-28

**Authors:** Cai Ye, Yijie Liu, Yue Li, Zan Li, Liyuan Sui, Jiwen Cui, Zihao Jiang, Jinlian Li, Jianjun Song, Jiguang Liu

**Affiliations:** 1 Key Laboratory of Microecology-immune Regulatory Network and Related Diseases School of Basic Medicine, Jiamusi University, Jiamusi, Heilongjiang, China; 2 College of Pharmacy, Jiamusi University, Jiamusi, Heilongjiang, China

**Keywords:** atopic dermatitis, NF-κB, *Schisandra chinensis* stem, Schisandrin B, TRPV1

## Abstract

**Background:**

Atopic dermatitis (AD) is a chronic, pruritic, inflammatory skin disorder. While the stem of Schisandra chinensis has been extensively studied for its pharmacological properties, including anti-inflammatory, antioxidant, and hepatoprotective effects, its therapeutic potential in AD remains to be elucidated. This study therefore aimed to investigate the effects of *Schisandra chinensis* stem extract (SCSE) against AD and to explore its underlying mechanism of action.

**Methods:**

Chemical profiling of SCSE via UPLC-Q-Exactive-Orbitrap-MS revealed 45 constituents, with lignans comprising 80%. The primary active component was identified through activity-guided assays employing hyaluronidase inhibition and HPLC. The therapeutic efficacy of SCSE and its constituent Schisandrin B (Sch B) was assessed in an AD mouse model. Furthermore, network pharmacology predicted the involved signaling pathways, and these predictions were subsequently validated experimentally.

**Results:**

Sch B was identified as the core active component. Both SCSE and Sch B significantly improved skin barrier function in AD mice, as evidenced by reduced transepidermal water loss (TEWL) and upregulation of key barrier proteins (Filaggrin, Loricrin, and Claudin-1). They also alleviated pruritus by suppressing Transient Receptor Potential Vanilloid 1 (TRPV1) and mitigated the allergic-inflammatory response, as shown by reduced Immunoglobulin E (IgE) levels and inhibited release of mast cell (MC) mediators (IL-4, IL-6, TNF-α). These effects were potentially mediated through modulation of the NF-κB pathway.

**Conclusion:**

By simultaneously mitigating skin barrier dysfunction, immune inflammation, and pruritus, SCSE and Sch B hold promise as therapeutic candidates capable of disrupting the self-perpetuating cycle of AD. These findings position SCSE and Sch B as a novel therapeutic strategy for this disease.

## 1 Introduction

Atopic dermatitis (AD), a chronic and relapsing inflammatory skin condition, is characterized by impaired skin barrier function, persistent inflammation, and severe pruritus. Its global prevalence is on the rise, impacting 15%-30% of children and 2%-10% of adults. This substantial disease burden has led the World Health Organization to designate AD as a priority non-communicable disease, thereby establishing it as a pressing worldwide public health concern (1, 2). Currently, the therapeutic arsenal for AD includes corticosteroids, topical calcineurin inhibitors, antihistamines, and biologics. However, each of these options is associated with certain limitations and side effects. Long-term use of corticosteroids can lead to skin atrophy, while calcineurin inhibitors may cause a burning sensation upon application. Antihistamines are linked to risks of osteoporosis, osteonecrosis, arteriosclerosis, diabetes, and dyslipidemia with prolonged use. Furthermore, biologics are often prohibitively expensive, imposing a significant financial burden on patients requiring long-term therapy (3, 4). Therefore, developing new AD therapeutics that offer high efficacy, a favorable safety profile, and affordability is an urgent and unmet medical need.

The pathogenesis of AD is complex, driven by the interplay of immune dysregulation, barrier dysfunction, and neurogenic inflammation. However, its precise mechanisms are not yet fully understood (5–8). A core feature of AD is intense pruritus, which perpetuates a vicious cycle: scratching in response to itch aggravates skin lesions and further compromises the barrier, reinforcing the “barrier damage-immune inflammation-pruritus” feedback loop. Transient Receptor Potential Vanilloid 1 (TRPV1), the prototypical member of the vanilloid subfamily within the Transient Receptor Potential (TRP) channel superfamily, acts as a pivotal integrator of barrier, immune, and neural functions. Its activation induces calcium influx, leading to the release of neuropeptides from sensory neurons. This process ultimately promotes neurogenic inflammation and intensifies itching, thereby playing a pivotal role in AD pathogenesis (9). Upon engagement of surface-bound IgE with its high-affinity receptor (FcϵRI), mast cells (MCs)-key effector cells-undergo degranulation, releasing mediators such as histamine (His), tumor necrosis factor-α (TNF-α), and interleukin-31 (IL-31). These mediators directly stimulate sensory neurons and sensitize the TRPV1 channel by lowering its activation threshold, thereby establishing a “MCs-TRPV1-neuropeptide” circuit. Conversely, TRPV1 activation-induced calcium influx and the subsequent release of neuropeptides such as substance P (SP) can, in turn, reactivate mast cells, reinforcing this positive feedback loop (10–12). Furthermore, Nuclear Factor Kappa B (NF-κB), a master regulator of inflammation, is activated by both MC-derived mediators like TNF-α and TRPV1-mediated calcium signaling, leading to amplified production of pro-inflammatory cytokines and exacerbation of chronic inflammation (13, 14). Therefore, a strategy that concurrently targets MC activation and TRPV1 signaling not only directly alleviates pruritus and neurogenic inflammation but also, by inhibiting the downstream NF-κB pathway, effectively disrupts the “barrier dysfunction-immune inflammation-pruritus” vicious cycle. Consequently, such a multi-targeted approach against this core axis offers a highly promising strategy for AD treatment.

Currently, natural compounds are garnering increasing attention as potential alternatives to existing drugs. *Schisandra chinensis* (Turcz.) Baill. is a traditional herb in Chinese medicine. Its fruit, rich in lignans with demonstrated antioxidant, anti-inflammatory, and immunomodulatory properties, has been shown to significantly ameliorate skin thickening and inflammatory infiltration in AD-like mouse models (15, 16). However, the fruit requires years to mature and its harvest necessitates destruction of the plant, presenting significant limitations for sustainable resource utilization. Recent investigations have revealed that the stems of *Schisandra chinensis* are also abundant in lignans identical to those in the fruit, with levels of certain constituents exceeding those found in the fruit, implying a potential for superior anti-inflammatory and immunomodulatory efficacy (17). Notably, schisandrin B (Sch B), the principal active lignan in the stems, demonstrates a wide range of pharmacological activities, including hepatoprotective, antitumor, immunomodulatory, and marked antioxidant and anti-inflammatory properties (18, 19). The stems exhibit rapid regrowth, allow for sustainable harvesting with minimal damage to the plant, and are associated with low raw material costs. Furthermore, their low fiber content simplifies the extraction process. However, current research on the stem of *Schisandra chinensis* remains predominantly focused on hepatotoxicity, and its therapeutic potential for AD has not yet been validated.

This study was designed to systematically evaluate the active constituents of *Schisandra chinensis* stem extract (SCSE) and its therapeutic potential and mechanisms in AD. The chemical profile of SCSE was characterized qualitatively and quantitatively by employing UPLC-Q-Exactive-Orbitrap-MS and HPLC techniques, respectively. Furthermore, key anti-allergic constituents were identified via a hyaluronidase inhibition assay. The therapeutic effects of SCSE and Sch B were comprehensively evaluated through a series of key metrics: clinical severity scores (encompassing erythema, edema/papulation, excoriation, and lichenification), transepidermal water loss (TEWL), serum IgE levels, mast cell degranulation, and TRPV1 protein expression. The mechanism of action for SCSE was further elucidated and validated by integrating network pharmacology prediction with western blot analysis. Collectively, this work provides a scientific foundation for new AD drug development and significantly advances the potential for the comprehensive utilization of *Schisandra chinensis* stems.

## 2 Materials and methods

### 2.1 Drugs and reagents

Schisandrin B (purity >95%) and 2,4-dinitrochlorobenzene (DNCB, purity 99%) were purchased from PuSi Biological Technology Co., Ltd. (Chengdu, China). Reference standards—schisandrin B (purity ≥98%), schisandrol A (≥98%), schisandrol B (≥98%), schizandrin A(≥98%), schisandrin C (≥98%), and schisantherin A (≥98%)—as well as hyaluronidase and sodium hyaluronate, were obtained from Shanghai Yuanye Bio-Technology Co., Ltd. (Shanghai, China). The HPLC-grade methanol, acetonitrile, and formic acid were purchased from ThermoFisher (Waltham, MA, USA). The enzyme-linked immunosorbent assay (ELISA) kits for IgE (Andy Gene, E-20545), TNF-α(Andy Gene, E-20219), SP (Andy Gene, E-20436), histamine (Andy Gene, E-20451), tryptase (Andy Gene, E-21450), Interleukin-4 (IL-4) (Andy Gene, E-20011), Interleukin-6 (IL-6) (Andy Gene, E-20012), and IL-31 (Andy Gene, E-20667) were pur chased from the Enzyme-free Industry Co., Ltd. (Jiangsu, China). Antibodies against TRPV1 (ab6166), filaggrin (FLG, ab81468), loricrin (LOR, ab85679), Claudin-1 (ab15098), NF-κB p65 (ab16502), phospho-NF-κB p65 (ab86299), IKKα/β (ab178870), and phospho-IKKα/β (ab194528) were obtained from Abcam (Cambridge, UK). Antibodies against IκBα (A19714) and phospho-IκBα (AP0420) were obtained from ABclonal Technology (Wuhan, China).

### 2.2 Preparation of SCSE

The stems of *Schisandra chinensis* were collected from the Jiamusi region of Heilongjiang Province, China. The plant material was dried, pulverized, and passed through a 40-mesh sieve. The powdered stems were first steeped in 10 volumes of 70% ethanol for 12 h, followed by two rounds of ultrasonic-assisted extraction (70 °C, 250 W) with each session performed for 1 h. The combined extracts were sequentially processed by petroleum ether extraction, filtration, and concentration under reduced pressure. The resulting concentrate was lyophilized to obtain a dry powder of *Schisandra chinensis* stem extract (SCSE), which was stored at –80°C for subsequent experiments.

### 2.3 Qualitative and quantitative analysis of SCSE

#### 2.3.1 Qualitative profiling of SCSE

The chemical profile of SCSE was analyzed using a UPLC-Q-Exactive Focus system (Thermo Scientific) equipped with a Thermo Hypersil GOLD C18 column (1.9 μm, 100 mm × 2.1 mm). The column temperature was maintained at 35°C, and the injection volume was 2 μL. Separation was achieved using a gradient elution program with 0.1% formic acid in water (mobile phase A) and acetonitrile (mobile phase B) at a flow rate of 0.2 mL/min. The gradient program was as follows: 0–3 min, 23% B; 3–7 min, 23%-40% B; 7–8 min, 40%-42% B; 8-11.5 min, 42%-55% B; 11.5–13 min, 55%-65% B; 13-14.5 min, 65%-75% B; 14.5–19 min, 75%-100% B; 19–28 min, 100%-23% B. Detection was performed at 216 nm, and chromatograms were recorded over 30 min.

Mass spectrometric analysis was conducted using a heated electrospray ionization (HESI) source in both positive and negative ionization modes. The ion spray voltages were set to 3.8 kV for positive mode and 3.2 kV for negative mode. The sheath gas flow rate was 35 arb, auxiliary gas pressure was 5 psi, and the ion transfer tube temperature was maintained at 320°C. The HESI source temperature was set to 350°C. Full MS/ddMS2 scans were performed with a mass range of m/z 80–1200 Da, with full MS and dd-MS² resolutions set to 70,000 and 17,500, respectively.

#### 2.3.2 Quantification of lignan constituents

Reference standards of deoxyschisandrin, schisandrin B, gomisin N, schisandrol A, schisandrol B, and schisantherin A (10 mg each) were accurately weighed and individually dissolved in ethanol in 10 mL volumetric flasks. The solutions were vortex-mixed, filtered through 0.22 μm microporous membranes, and stored as individual stock solutions. A mixed standard solution was prepared by combining appropriate aliquots from each stock solution and subsequently diluted to a series of concentrations for constructing calibration curves (**Supplementary Figure 1**, **Table 1**).

**Table 1:** Chemical composition mass spectrometry information of SCSE extract.

No.	t_R_/min	Error	Adduct	Theoretical value	Detected value	Ion fragments	Formula	Name
1	0.91	0.486	[M-H]^-^	354	353.08749	266.14368, 292.12338 335.14154	C_16_H_17_O_9_	Neochlorogenic acid
2	1.03	0.738	[M-H]^-^	192	191.01877	173.00783, 154.99689	C_6_H_7_O_7_	Citric acid
3	1.23	-8.269	[M-H]^+^	302	303.04852	285.03870, 257.04303	C_15_H_10_O_7_	Quercetin
4	1.42	-11.551	[M-H]^+^	126	127.03860	69.07024, 61.02874	C_6_H_6_O_3_	3- hydroxymethyl -2- furfural
5	1.60	-2.974	[M-H]^-^	154	153.01778	109.02802, 91.01721	C_7_H_5_O_4_	Protocatechuic acid
6	2.60	-10.929	[M-H]^-^	122	121.02818	119.01274, 108.02016	C_7_H_5_O_2_	P-hydroxybenzaldehyde
7	5.61	-2.354	[M-H]^-^	280	279.12314	121.02808,108.02016	C_15_H_10_O_5_	Psilostachyin A
8	6.40	-4.502	[M-H]^-^	168	167.03423	152.01038,123.04373	C_8_H_8_O_4_	Vanillic acid
9	6.78	-3.090	[M-H]^-^	462	461.10751	433.07693, 313.03421	C_22_H_22_O_11_	Isosorbide
10	7.50	-0.617	[M-H]^-^	264	263.12872	245.11813, 219.13821	C_15_H_20_O_4_	abscisic acid
11	7.76	-9.958	[M-H]^+^	196	197.15274	179.05082, 161.09547	C_12_H_20_O_2_	Neryl acetate
12	8.29	-7.088	[M-H]^+^	430	431.20447	356.16006, 326.14886	C_24_H_30_O_7_	Schinsanlignone A
13	8.47	-7.233	[M-H]^+^	400.5	401.19406	401.19406, 401.19406	C_23_H_29_O_6_	Gomisin N
14	8.54	-8.164	[M-H]^+^	432	433.21964	384.19135, 369.16815	C_24_H_32_O_7_	Sschisandrol A
15	9.24	-4.719	[M-H]^+^	358.4	359.14722	327.15695, 327.15695	C_20_H_22_O_6_	Pinoresinol
16	10.01	-7.158	[M-H]^+^	400	401.19409	386.17130, 309.14508	C_23_H_28_O_6_	Schisandrin B
17	10.26	-14.446	[M-H]^+^	500	500.23434	499.26334, 401.19421	C_28_H_36_O_8_	Angeloylgomisin H
18	11.21	3.425	[M-H]-	420	419.17148	278.15460, 197.11685	C_22_H_26_O_8_	Syringaresinol
19	11.45	-7.379	[M-H]^+^	388	389.19409	287.08984, 227.06909	C_22_H_28_O_6_	Gomisin J
20	12.30	2.677	[M-H]^-^	270	269.04517	241.05042, 225.05493	C_15_H_10_O_5_	Apigenin
21	12.50	-7.005	[M-H]^+^	500	501.24588	370.17590, 337.14209	C_28_H_36_O_8_	Tigloylgomisin H
22	12.50	-4.656	[M-H]^+^	514.56	515.22516	385.16281, 298.08200	C_28_H_34_O_9_	Gomisin F
23	12.50	-6.785	[M-H]^+^	514.56	515.22516	385.16281, 316.09235	C_28_H_34_O_9_	Tigloylgomisin P
24	12.50	-6.785	[M-H]^+^	514.6	515.22516	385.16281, 316.09235	C_28_H_34_O_9_	Schisantherin B
25	12.74	-7.316	[M-H]^+^	390	391.20975	167.06953, 121.06451	C_22_H_30_O_6_	Pregomisin
26	12.97	1.662	[M-H]^+^	416	417.19257	354.17691, 284.20386	C_23_H_28_O_7_	Schisandrol B
27	13.08	-7.896	[M-H]^+^	522	523.22961	523.22961, 523.22961	C_30_H_34_O_8_	Benzoylgomisin H
28	13.30	-5.997	[M-H]^+^	530	531.25677	383.14716, 352.12885	C_29_H_38_O_9_	Angeloylgomisin Q
29	13.64	-4.499	[M-H]^+^	402.5	401.19406	340.16470, 370.17548, 386.17072	C_23_H_30_O_6_	Schisanhenol
30	13.66	-11.124	[M-H]^+^	536	537.20703	366.14435, 384.19070, 415.20596	C_30_H_32_O_9_	Schisantherin A
31	13.66	-9.082	[M-H]^+^	536.57	537.20703	491.20169, 407.14423	C_30_H_32_O_9_	Gomisin G
32	13.73	-7.262	[M-H]^+^	386	387.17850	355.15253, 325.14136	C_22_H_26_O_6_	Gomisin R
33	13.73	-7.262	[M-H]^+^	386	387.17850	355.15253, 325.14136	C_22_H_26_O_6_	Gomisin M2
34	14.00	-7.170	[M-H]^+^	384	385.16290	343.15225, 280.10767	C_22_H_24_O_6_	Schisandrin C
35	14.29	-8.108	[M-H]+	328.4	327.15753	295.13022, 203.10585	C_20_H_22_O_4_	(+)-anwulignan
36	14.33	-8.474	[M-H]^+^	326	327.15741	295.13022, 284.10150	C_20_H_22_O_4_	LicarinA
37	14.42	-6.374	[M-H]^+^	416	415.17358	346.13928, 328.12869	C_23_H_28_O_7_	Gomisin O
38	14.42	-6.374	[M-H]^+^	416.5	415.17358	346.13928, 328.12869	C_23_H_28_O_7_	Gomisin A
39	14.45	-4.455	[M-H]^+^	416	415.17328	346.13928, 328.12869	C_23_H_28_O_7_	Epigomisin O
40	14.96	-6.785	[M-H]^+^	514.57	515.22516	385.16281, 298.08200	C_28_H_34_O_8_	Angeloylgomisin P
41	15.10	-5.751	[M-H]^+^	498	499.23087	468.24622, 401.19421	C_28_H_34_O_8_	Angeloylgomisin O
42	15.50	-7.003	[M-H]^+^	416	417.22534	370.17599, 316.12894	C_24_H_32_O_6_	Schizandrin A
43	15.88	-8.854	[M-H]^+^	470	471.34381	453.32980, 435.32108	C_30_H_46_O_4_	Kadsuric acid
44	15.99	-9.621	[M-H]^+^	204	205.19420	163.14722, 121.10084	C_15_H_24_	Calarene
45	26.06	0.391	[M-H]^-^	470	469.33142	451.31949, 425.34177	C_30_H_46_O_4_	Nigranoic acid

The quantitative analysis of six major lignans in *Schisandra chinensis* stem extract (SCSE) was performed using a Shimadzu high-performance liquid chromatography (HPLC) system. Separation was achieved on a C18 column (4.6 × 250 mm, 5 μm) maintained at 30°C. The mobile phase consisted of 0.1% formic acid in water (A) and acetonitrile (B), with a gradient elution program as follows: 0–16 min (48% B), 16–24 min (55% B), 24–35 min (60% B), 35–40 min (75% B), 40–50 min (60% B), and 51–55 min (48% B). The flow rate was set at 1.0 mL/min, the injection volume was 15 μL, and detection was carried out at 256 nm. The column was re-equilibrated under initial gradient conditions for 5 min between consecutive runs. A representative chromatogram of the mixed standard solution is provided in **Supplementary Figure 2** (20).

### 2.4 Hyaluronidase inhibition assay

The hyaluronidase inhibitory activity was evaluated with minor modifications according to Gavini et al. (21). Briefly, 50 μL of each test sample solution was mixed with 50 μL of hyaluronidase solution (0.3 mg/mL) and incubated at 37°C for 10 min. Subsequently, 12 μL of phosphate buffer (0.3 mol/L, pH 5.35) was added, followed by another 10 min incubation at 37°C. Then, 50 μL of sodium hyalur-onate solution was added, and the mixture was incubated for 45 min at 37°C. After the addition of 100 μL of acidic albumin solution, the reaction system was incubated at room temperature for 10 min to facilitate the formation of a precipitate between undegraded hyaluronate and albumin. The absorb-ance was measured at 600 nm, and all assays were performed in triplicate.


$$Hyaluronidase\;inhibition\;rate\%=\frac{OD_{sample}-OD_{control}}{OD_{control}}\times 100\%$$

Here, OD_sample_ refers to the optical density of the sample extract, and OD_control_ refers to the optical density measured using buffer instead of the sample.

### 2.5 Induction and treatment of AD-like skin lesions in mice

All animal experimental procedures were approved by the Animal Ethics Committee of the College of Pharmacy, Jiamusi University, China (Permit No. JDYXY-2024014) and were conducted in strict accordance with the “Regulations for the Administration of Affairs Concerning Experimental Animals” issued by the State Council of the People’s Republic of China.

Male BALB/c mice (5–6 weeks old) were randomly assigned to nine experimental groups (n = 6 per group): Group 1, normal control (untreated); Group 2, DNCB control (model group); Group 3, DNCB + 15 mg/kg dexamethasone (Dexa); Groups 4-6, DNCB + SCSE (200, 400, and 600 mg/kg; designated as SCSE-Low, -Middle, and -High, respectively); Groups 7-9, DNCB + Sch B (200, 400, and 600 mg/kg; designated as Sch B-Low, -Middle, and -High, respectively). The dosages of SCSE and Sch B were determined based on preliminary experiments. The dosages of SCSE and Sch B were determined based on preliminary experiments. An acute toxicity test showed no adverse effects at doses up to 2000 mg/kg for SCSE and 1000 mg/kg for Sch B, establishing a wide safety margin. Based on the effective range identified in pilot dose-ranging studies, the formal experiments employed doses of 200, 400, and 600 mg/kg. These doses are consistent with those commonly used for natural extracts in murine AD models.

The mouse model of atopic dermatitis was induced, with minor modifications, according to the method previously described by An et al. (22). On day 0 of the sensitization phase, the dorsal hair of all mice was shaved. On day 1, all groups except the normal control were topically sensitized with 150 µL of 2% DNCB applied to the shaved area. The DNCB solution was prepared in a vehicle of acetone and olive oil (3:1, V/V). The normal control group received an equal volume of the vehicle alone. From day 5 to day 21, all groups except the normal control were challenged by topical application of 0.5% DNCB solution to the dorsal skin every three days to induce dermatitis (challenge phase). The normal control group continued to receive an equal volume of the vehicle during each challenge. From day 1 to day 20, all groups were administered 0.2 mL of their respective test compounds suspended in 0.5% sodium carboxymethyl cellulose (CMC-Na) solution daily by oral gavage. The normal control and DNCB model groups received 0.2 mL of the 0.5% CMC-Na vehicle only. On day 21, dorsal skin tissues, dorsal root ganglia (DRG), and blood samples were collected. Blood samples were centrifuged at 3,000 × g for 10 min to obtain serum. The serum, DRG, and portions of the skin samples were snap-frozen and stored at −80°C for subsequent analysis. The remaining skin tissues were fixed in 4% paraformaldehyde for 24 h at 4°C for histopathological examination.

### 2.6 Assessment of dermatitis severity and scratching behavior

Dermatitis severity and scratching behavior were evaluated according to the method described by Kim et al. with minor modifications (23). Specifically, dermatitis severity was evaluated weekly using a scoring system that assessed four clinical signs: edema, erythema/hemorrhage, erosion/excoriation, and scaling. Each sign was graded on a scale of 0 to 3 (0 = none, 1 = mild, 2 = moderate, 3 = severe), and the total dermatitis severity score was defined as the sum of the scores for these four symptoms. For scratching behavior, mice were recorded for 10 minutes using a digital camera. A scratching bout was defined as a series of consecutive scratching movements directed at the dorsal skin with the hind paw. The total number of bouts during the 10-minute period was manually counted by a observer blinded to group allocation.

### 2.7 Histopathological examination

Dorsal skin tissues from six mice per group were fixed in 4% paraformaldehyde, embedded in paraffin, and sectioned at a thickness of 4 μm. Following deparaffinization, sections were stained with hematoxylin and eosin (H&E) to evaluate epidermal hyperplasia (thickness) and with toluidine blue to assess mast cell infiltration. For each section, three random fields were selected, and the mean epidermal thickness and mast cell count were calculated. All sections were imaged under a microscope (BX50F; Olympus Optical Co., Ltd.), and image analysis was performed using Image Pro Plus Version 4.5 software (Media Cybernetics Co., Silver Spring, MD, USA).

### 2.8 ELISA

Serum levels of IgE, histamine, TNF-α, IL-4, IL-6, and SP were measured using commercial ELISA kits according to the manufacturers’ instructions. Tryptaselevels in lesional skin tissues were also quantified by ELISA following tissue homogenization in phosphate-buffered saline.

### 2.9 Western blot analysis

Total proteins were extracted from mouse dorsal skin tissues using RIPA lysis buffer (Beyotime, China) supplemented with protease and phosphatase inhibitors. Protein concentrations were determined with a BCA assay kit (Beyotime, China). Equal amounts of protein (30 μg per lane) were separated by 12% SDS−PAGE and transferred onto PVDF membranes (Millipore, USA). After blocking with 5% non−fat milk in TBST, membranes were incubated overnight at 4°C with primary antibodies (see section 2.1) and then with an HRP−conjugated goat anti−rabbit secondary antibody for 1 h at room temperature. Signals were detected using ECL reagent (Boster, China) and quantified with ImageJ software (NIH); β−actin (or GAPDH) served as the loading control.

### 2.10 Network pharmacology and molecular docking

The network pharmacology study was conducted according to the method described by Liu et al. with minor modifications (24). The potential targets of Sch B were predicted using the SwissTargetPrediction database (http://www.swisstargetprediction.ch/) and the PharmMapper platform (https://www.lilab-ecust.cn/pharmmapper/). Disease-associated targets for atopic dermatitis (AD) were retrieved from the OMIM (https://omim.org/) and GeneCards (https://www.genecards.org/) databases. The intersection between Sch B-related targets and AD-related targets was identified and visualized as a Venn diagram, yielding a set of common targets for further analysis. The common targets were imported into the STRING database (https://string-db.org/) to construct a protein-protein interaction (PPI) network. Network topology analysis was performed using Cytoscape software (version 3.9.1; https://cytoscape.org/) to identify core targets. Gene Ontology (GO) enrichment analysis (including biological process, cellular component, and molecular function) and Kyoto Encyclopedia of Genes and Genomes (KEGG) pathway enrichment analysis were conducted for the key targets through the Metascape database (https://metascape.org) to elucidate the potential biological processes and signaling pathways involved in Sch B treatment of AD.

The two-dimensional structure of Sch B was obtained from the PubChem database (https://pubchem.ncbi.nlm.nih.gov/), converted to a three-dimensional structure using Chem3D, and energy minimization was performed. The three-dimensional crystal structures of core target proteins were downloaded from the RCSB PDB database (https://www.rcsb.org/), and irrelevant molecules such as water molecules and non-protein ligands were removed using PyMOL software. Semi-flexible molecular docking was carried out with AutoDock Vina software (https://vina.scripps.edu/), where the docking box was set to cover the protein active pocket to evaluate the binding affinity between Sch B and the target proteins. The molecular docking results were analyzed for three-dimensional and two-dimensional interactions using PyMOL (https://pymol.org/) and Discovery Studio (https://www.3ds.com/products/biovia/discovery-studio) to visually present the binding modes.

### 2.11 Transepidermal water loss detection

TEWL was measured using the Tewameter^®^ TM 300 probe (Courage + Khazaka, Germany) to evaluate the integrity of the skin barrier function. During measurement, the probe was placed vertically and gently on the prepared skin surface, ensuring stable contact between the probe and the skin. When the reading stabilized (typically after 30–60 seconds), the TEWL value was recorded in grams per square meter per hour (g/m²/h). Three consecutive readings were taken at each measurement point, and the mean value was calculated as the final TEWL value for that point. After each animal was tested, the probe sensor was immediately wiped with a dedicated cleaning wipe to prevent cross−contamination between samples and to ensure measurement accuracy.

### 2.12 Determination of skin stratum corneum hydration

Skin hydration of the stratum corneum was measured using a Corneometer^®^ CM 825 probe (Courage + Khazaka, Germany). Prior to measurement, the dorsal skin of the mice was shaved and cleaned. During measurement, the probe was placed perpendicularly and gently in contact with the shaved area under constant pressure. The relative hydration value of the stratum corneum was directly recorded in arbitrary units (a.u.) as displayed by the instrument. Each measurement point was assessed three times, with an interval of at least 30 seconds between consecutive readings. The final hydration level for each site was expressed as the mean of the three measurements.

### 2.13 Cell culture

The rat basophilic leukemia cell line RBL-2H3 was obtained from the Cell Bank of the Chinese Academy of Sciences and cultured in Minimum Essential Medium (MEM) supplemented with 10% fetal bovine serum (FBS), 100 U/mL penicillin, and 100 µg/mL streptomycin at 37°C in a humidified atmosphere containing 5% CO_2_.

### 2.14 Immunofluorescence staining

To assess NF-κB nuclear translocation, RBL-2H3 cells were first sensitized with 0.5 µg/mL DNP-IgE (Sigma-Aldrich, USA) overnight. The following day, cells were washed twice with PBS or culture medium to remove unbound IgE. Cells were then pretreated with SCSE (10 µg/mL) or Sch B (10 µg/mL) for 3 h. Subsequently, activation was induced by challenging the cells with 100 ng/mL DNP-BSA (Sigma-Aldrich, USA) for 2 h. Subsequently, cells were fixed with 4% paraformaldehyde (Beyotime, P0099), permeabilized with 0.1% Triton X-100 (Beyotime, ST675), and blocked with 5% BSA (Beyotime, P0007). Cells were then incubated overnight at 4°C with a rabbit anti-NF-κB p65 antibody (1:300). After washing, cells were incubated with an Alexa Fluor 594-conjugated goat anti-rabbit IgG secondary antibody (1:1000) for 1 h at room temperature in the dark. Nuclei were counterstained with DAPI. Fluorescent images were captured using a laser scanning confocal microscope (Nexcope NCF950) at 400× magnification. NF-κB p65 fluorescence intensity and its nuclear translocation were analyzed using ImageJ software.

### 2.15 Statistical analyses

All statistical analyses were performed using GraphPad Prism software (version 9; GraphPad Software, Inc., San Diego, CA, USA). Data are presented as the mean ± standard deviation (SD). Normality was assessed using the Shapiro-Wilk test. Comparisons between two groups were performed with an unpaired two-tailed Student’s t-test. Comparisons among multiple groups were analyzed by one−way analysis of variance (ANOVA) followed by Dunnett’s post−hoc test. A p−value< 0.05 was considered statistically significant. For *in vivo* experiments, the sample size *n* refers to the number of biological replicates (individual animals per group). For *in vitro* assays, *n* indicates the number of technical replicates. All experiments were independently repeated at least three times.

## 3 Results

### 3.1 Analysis of lignan constituents in SCSE and screening of active components

**Figure 1:**
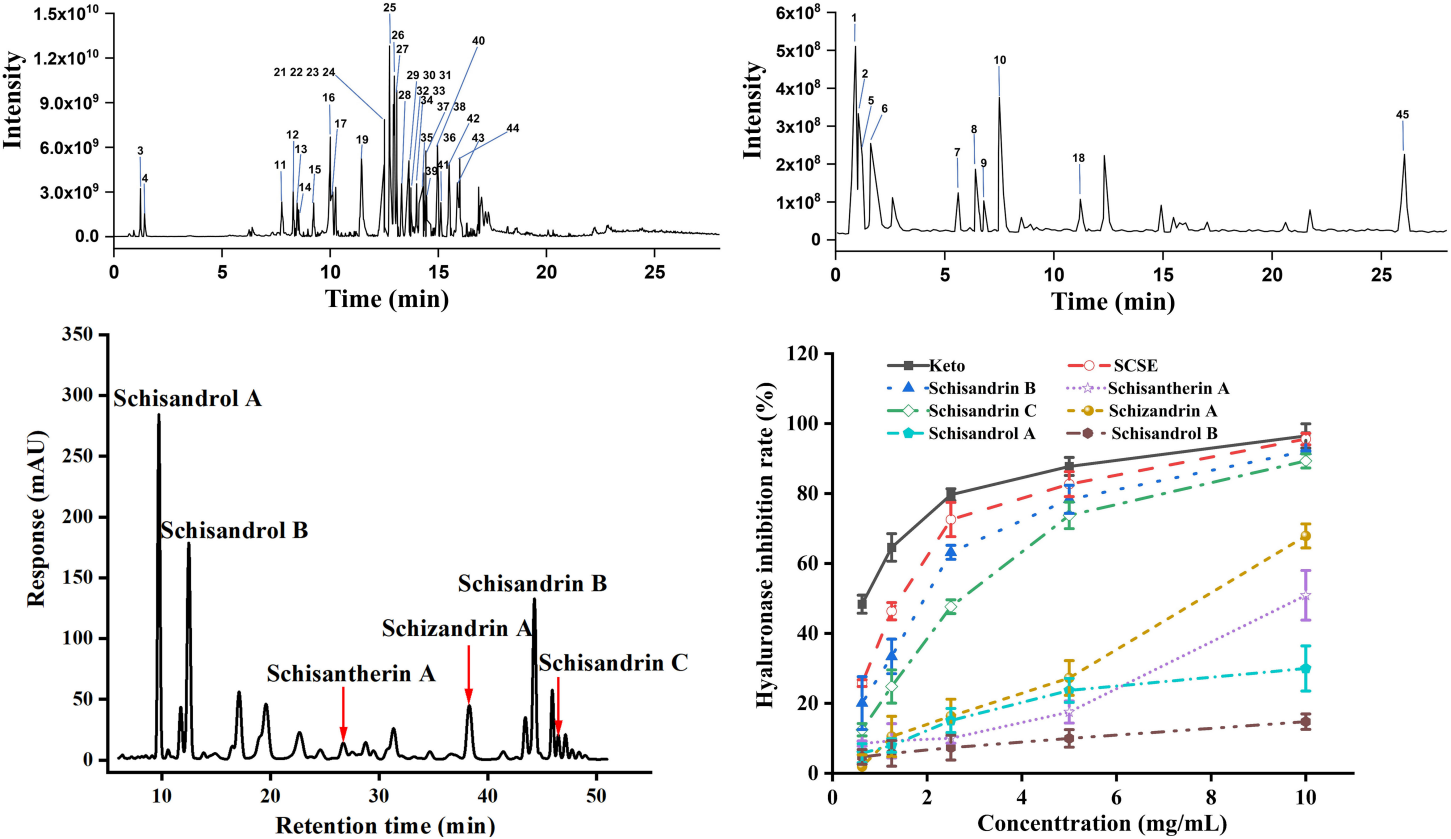
The total ion chromatograms of extract from the stems of schisandra chinensis (SCSE). **(A)** Positive ion mode. **(B)** Negative ion mode. **(C)** HPLC hromatogram of SCSE. **(D)** Hyaluronase inhibition rates of SCSE. Data are presented as mean ± SD (n = 3 technical replicates).

Qualitative analysis of *Schisandra chinensis* stem extract (SCSE) was performed using UPLC-Q-Exactive-Orbitrap-MS. Total ion chromatograms (TICs) of SCSE were obtained in both positive and negative ionization modes (**Figures 1A, B**). Analysis using the Compound Discoverer 3.0 platform led to the identification and characterization of 45 chemical constituents in SCSE. Lignans represented the predominant class, accounting for approximately 80% of the characterized compounds, while the remaining components included organic acids, flavonoids, and sesquiterpenoids (**Table 1**). Furthermore, the contents of six major lignans in SCSE were quantified by high-performance liquid chromatography (HPLC) (25). The quantitative results were as follows: schisandrol B (61.68 ± 0.085 mg/g), schisandrol A (56.86 ± 0.033 mg/g), schisandrin B (45.86 ± 0.801 mg/g), schizandrin A (21.73 ± 0.05 mg/g), schisandrin C (16.48 ± 0.116 mg/g), and schisantherin A (10.09 ± 0.274 mg/g) (**Figure 1C**). Structures of the six major lignans are shown in **Supplementary Figure 3**.

The hyaluronidase inhibition assay was employed as a standard *in vitro* method to evaluate anti-allergic activity. The inhibitory activities of SCSE and its six major lignans were tested, which revealed that SCSE exhibited significant hyaluronidase inhibition. Notably, schisandrin B (Sch B) demonstrated inhibitory efficacy comparable to that of the complete SCSE (**Figure 1D**). These findings establish Sch B as the critical active component underlying the anti-allergic properties of SCSE.

### 3.2 SCSE and Sch B ameliorated DNCB-induced AD-like symptoms and restored skin barrier function in mice

**Figure 2:**
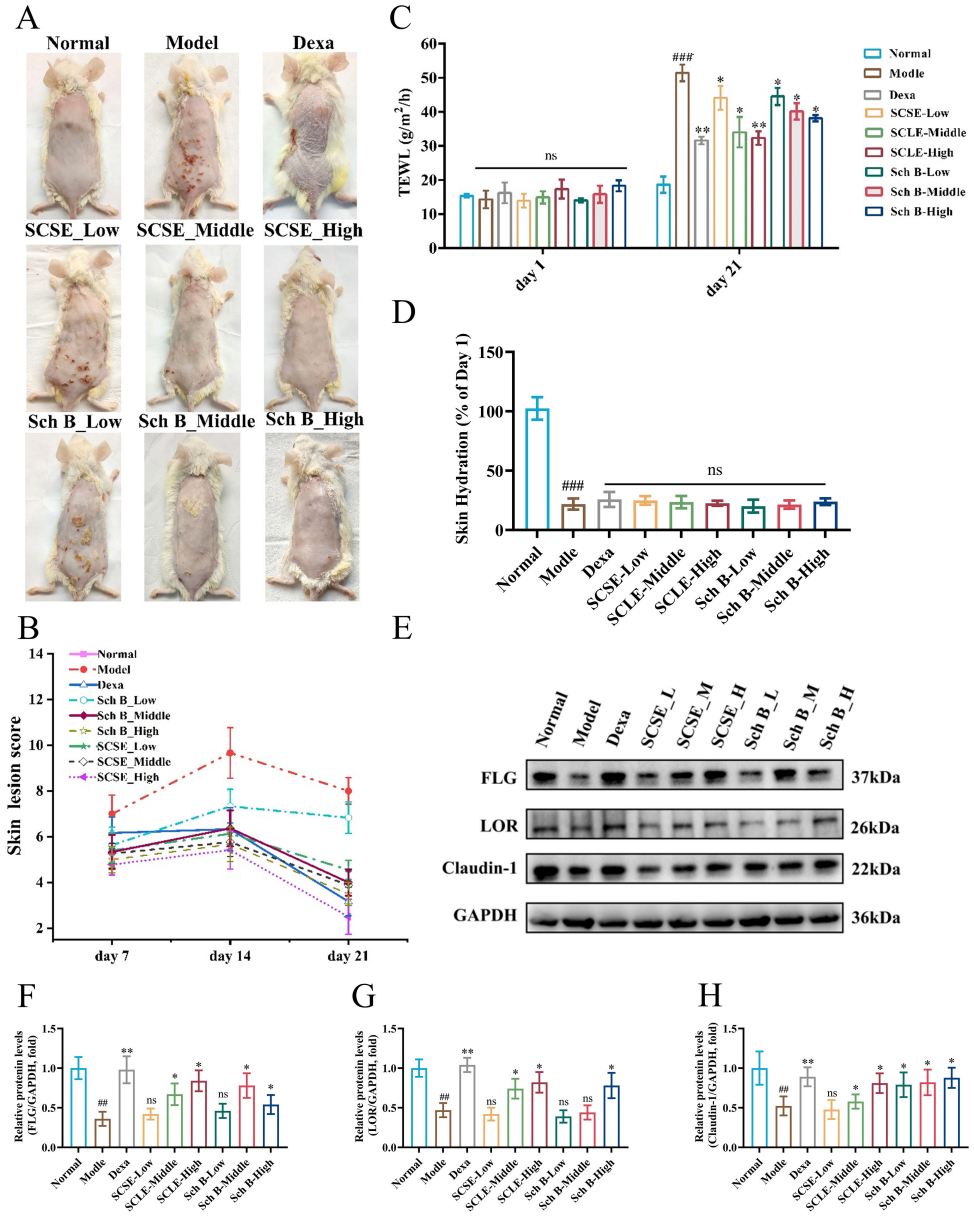
SCSE and Sch B ameliorated DNCB-induced AD-like symptoms and restored skin barrier function in mouse. **(A)** Photographs for the clinical features of mouse dorsal skin lesion. **(B)** Changes in mean clinical skin severity score. Analysis of the effect of the change in trans-epidermal water loss (TEWL) **(C)** and hydration **(D)** in an atopic-dermatitis-like phenotype in BALB/c mice. The protein levels of FLG, LOR and claudin-1 in skin, **(E)** Western blot, **(F–H)** quantitative analysis of band intensity. Actin was used as a reference protein. Data are presented as mean ± SD (n = 6 biological replicates). Differences between the control and model groups were analyzed by an unpaired two-tailed Student’s t-test. Comparisons among the model group and treatment groups were analyzed by one−way ANOVA followed by Dunnett’s test. Significance levels are indicated as: ^##^ p < 0.01, ^###^ p < 0.001 vs. the control group; *p < 0.05, **p < 0.01 vs. the model group. ns, not significant.

In this study, we assessed the restorative effects of SCSE and Sch B on epidermal barrier integrity in a DNCB-induced AD mouse model through a comprehensive evaluation, which included: (i) dermatitis severity scores (**Figures 2A, B**); (ii) skin barrier function parameters, including transepidermal water loss (TEWL) and skin hydration levels (**Figures 2C, D**); and (iii) the expression levels of key barrier proteins, including filaggrin (FLG), loricrin (LOR), and Claudin-1 (**Figures 2E–H**).

In the DNCB-induced AD mouse model, characteristic dermatitis phenotypes were observed, including erythema, skin thickening, sclerosis, and scaling. These manifestations were accompanied by significantly elevated inflammation scores, increased TEWL, reduced skin hydration, and downregulated expression of key barrier proteins (FLG, LOR, and Claudin-1), indicating impaired barrier function. Following treatment with different concentrations of SCSE and Sch B, both the skin lesion appearance and inflammation scores were ameliorated to varying degrees. Skin barrier function was significantly restored, as evidenced by markedly reduced TEWL, although the treatments showed limited effects on skin hydration. Concurrently, the protein expression levels of FLG, LOR, and Claudin-1 were significantly upregulated. These effects were consistent with the positive control drug dexamethasone (Dexa). These findings demonstrate that SCSE and Sch B alleviated AD-like dermatosis through a mechanism involving the upregulation of barrier protein expression, leading to reduced transepidermal water loss and ultimately the repair of DNCB-induced epidermal barrier disruption.

### 3.3 Network pharmacology analysis reveals potential targets and pathways for Sch B in the treatment of AD

**Figure 3:**
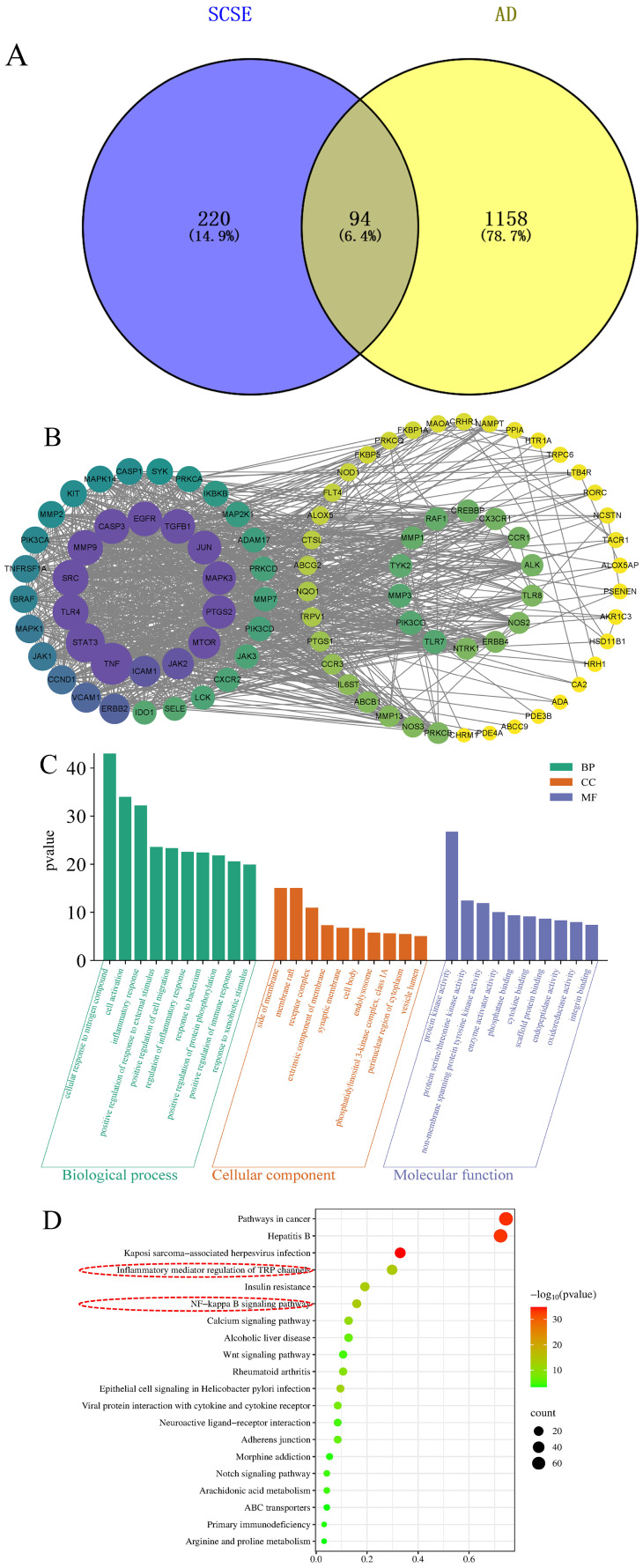
Network construction and analysis of Sch B treatment for AD. **(A)** Common targets between Sch B and AD. **(B)** Optimized PPI network. **(C)** The top 10 significantly enriched terms in GO functional enrichment analysis. **(D)** The top 20 significantly enriched terms in KEGG pathway enrichment analysis.

Target prediction for Sch B was performed using the PharmMapper and SwissTargetPrediction databases, yielding 314 potential targets. Disease-associated targets for atopic dermatitis (AD) were retrieved from the GeneCards and OMIM databases, resulting in 1,158 AD-related targets. By comparing the Sch B-related targets with the AD-related targets, 94 overlapping targets were identified and considered as potential therapeutic targets for Sch B in the treatment of AD (**Figure 3A**). A protein-protein interaction (PPI) network was constructed based on data retrieved from the STRING database and visualized using Cytoscape software, illustrating the interactive relationships among the targets (**Figure 3B**). Gene Ontology (GO) enrichment analysis indicated that the key biological processes primarily involved inflammatory response, response to external stimuli, and regulation of protein phosphorylation (**Figure 3C**). Kyoto Encyclopedia of Genes and Genomes (KEGG) pathway enrichment analysis revealed that the major signaling pathways included inflammatory mediator regulation of TRP channels, NF-κB signaling pathway, calcium signaling pathway, Notch signaling pathway, and Wnt signaling pathway (**Figure 3D**). Among these, the inflammatory mediator regulation of TRP channels and the NF-κB signaling pathway exhibited the highest enrichment scores and may play central roles in Sch B-mediated effects on AD. Notably, TRPV1 protein expression, associated with the TRP channel pathway, has been experimentally validated in both skin tissues and dorsal root ganglia.

### 3.4 SCSE and Sch B alleviate DNCB-induced skin pathological damage and suppress scratching behavior in AD mice

**Figure 4:**
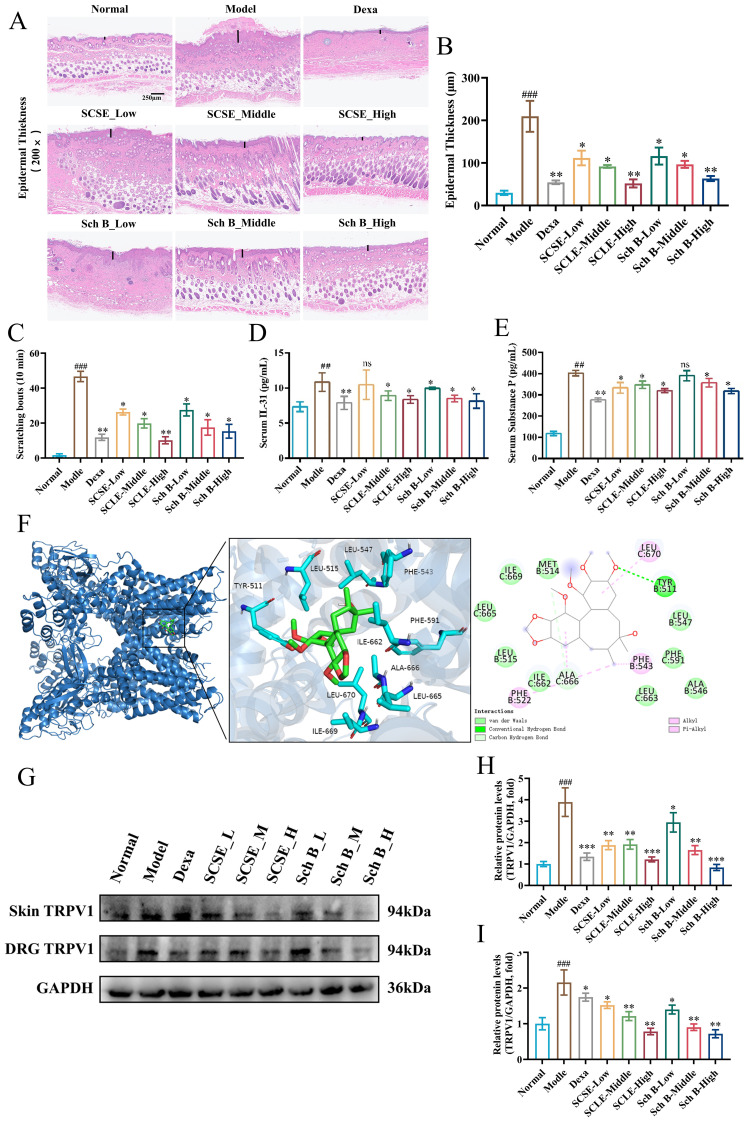
SCSE and Sch B attenuated DNCB-induced skin pathology and the associated scratching behavior in AD mice. **(A)** Skin tissues were stained with H&E to measure the thickness. **(B)** Epidermal thickness observed through H&E staining. **(C)** The scratching behavior of each mouse was observed for 10min after 3 weeks of drug application. **(D)** Levels of total IL-31 in serum **(E)** Levels of total SP in serum. **(F)** Molecular models of Sch B binding to the proteins of TRPV1. The protein levels of TRPV1 in skin and DRG, respectively. **(G)** western blot analysis of TRPV1 protein levels in skin and DRG, **(H)** and **(I)** quantitative analysis of band intensity, Actin was used as a reference protein. Data are presented as mean ± SD (n = 6 biological replicates). Differences between the control and model groups were analyzed by an unpaired two-tailed Student’s t-test. Comparisons among the model group and treatment groups were analyzed by one−way ANOVA followed by Dunnett’s test. Significance levels are indicated as: ^##^ p < 0.01, ^###^ p < 0.001 vs. the control group; * p < 0.05, **p < 0.01, *** p < 0.001 vs. the model group. ns, not significant.

To determine whether SCSE and Sch B suppress inflammatory cell infiltration and pathological changes in the DNCB-induced AD-like skin lesion model, skin tissue sections were subjected to hematoxylin and eosin (H&E) and toluidine blue staining. H&E staining was used to evaluate general histopathological alterations and inflammatory cell infiltration, while toluidine blue staining specifically identified mast cell infiltration. H&E staining revealed that mice in the model group exhibited epidermal thickening, hyperkeratosis, and substantial inflammatory cell infiltration. In contrast, SCSE- and Sch B-treated mice showed significantly reduced epidermal thickness and decreased inflammatory cell infiltration. The high-dose SCSE group demonstrated efficacy comparable to the Dexa group (**Figures 4A, B**). Pruritus is a core symptom of atopic dermatitis (AD). Behavioral assessment revealed that mice treated with SCSE or Sch B showed a significant reduction in scratching bouts compared to the model group (**Figure 4C**), indicating potent anti-pruritic efficacy. Furthermore, SCSE and Sch B significantly decreased serum levels of the pruritogenic factors IL-31 and SP, which were elevated in the model group (**Figures 4D, E**).

TRPV1 is a key mediator of pruritus in AD, directly activating itch-signaling neurons. Molecular docking predicted that Sch B could bind to TRPV1 (**Figure 4F**). Consistent with this prediction, Western blot analysis confirmed that SCSE and Sch B downregulated TRPV1 protein expression in lesional skin and dorsal root ganglia (DRG) (**Figures 4G–I**). These findings indicate that the downregulation of TRPV1, a principal target in pruritus, occurs not only within DRG sensory neurons and cutaneous nerve endings but also in local skin cells, including epidermal keratinocytes and infiltrating immune cells, correlating with the alleviation of pruritus in treated mice. The precise link between predicted binding and reduced expression, particularly regarding the specificity of action and the underlying mechanism, remains to be elucidated. Furthermore, functional validation of channel inhibition, such as through calcium imaging assays, was not performed in this study. Collectively, these data indicate an association between TRPV1 downregulation and the anti-pruritic effects of SCSE/Sch B, while direct evidence for functional channel modulation awaits further investigation.

TRPV1, a key driver of pruritus in AD, directly activates itch-signaling neurons. Molecular docking analysis indicated that Sch B stably binds to TYR511 and ALA666 residues of TRPV1 via hydrophobic interactions and hydrogen bonds, with a binding free energy of -6.7 kcal/mol (**Figure 4F**). Western blot analysis confirmed the significant downregulation of TRPV1 protein expression by both SCSE and Sch B in the lesional skin and dorsal root ganglia (DRG) of AD mice (**Figures 4G–I**). Collectively, these findings demonstrate that the considerable anti-allergic and anti-pruritic potential of SCSE and Sch B is closely associated with the modulation of the TRPV1 Channel.

### 3.5 SCSE and Sch B suppress mast cell degranulation and reduce the release of inflammatory factors

**Figure 5:**
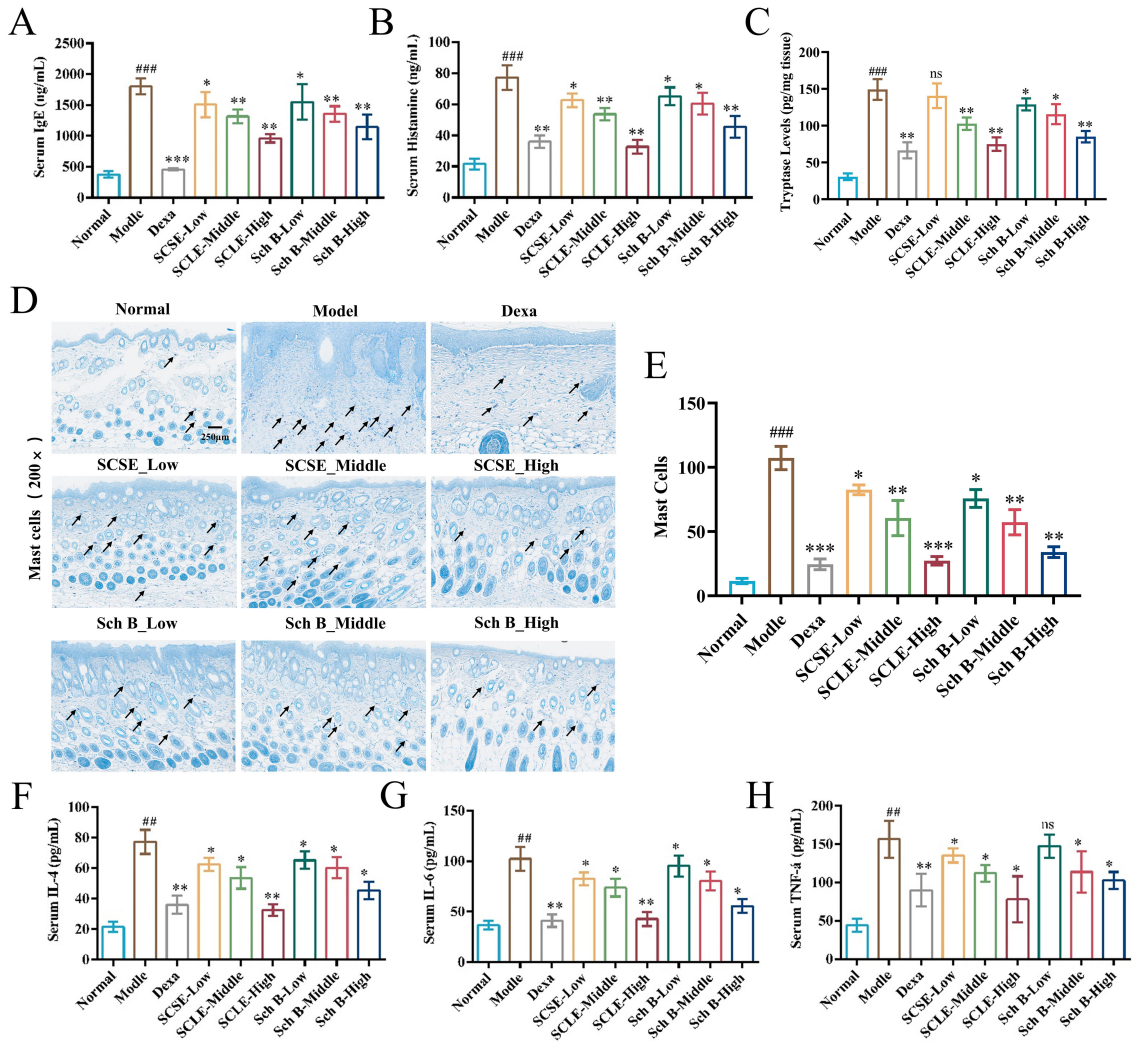
SCSE and Sch B inhibit mast cell degranulation and reduce the release of inflammatory factors. **(A)** Levels of total IgE in serum. **(B)** Levels of Histamine in serum. **(C)** Levels of Tryptase in skin tissues. **(D)** Representative images of toluidine blue staining for mast cells. **(E)** Quantification of the number of infiltrated mast cells (indicated by black arrows in D).. **(F)** Levels of IL-4 in serum. **(G)** Levels of IL-6 in serum. **(H)** Levels of TNF-α in serum. Values are mean ± SD (n = 6). Data are presented as mean ± SD (n = 6 biological replicates). Differences between the control and model groups were analyzed by an unpaired two-tailed Student’s t-test. Comparisons among the model group and treatment groups were analyzed by one−way ANOVA followed by Dunnett’s test. Significance levels are indicated as: ^##^ p < 0.01, ^###^ p < 0.001 vs. the control group; * p < 0.05, **p < 0.01, *** p < 0.001 vs. the model group. ns, not significant.

Elevated serum total IgE levels serve as a key biomarker of allergic responses during AD pathogenesis. The results showed that serum IgE levels were significantly increased in the model group, which promoted mast cell degranulation via IgE-FcϵRI cross-linking, leading to the release of histamine and tryptase, and subsequently driving the secretion of inflammatory factors including IL-4, IL-6, and TNF-α. However, compared with the model group, SCSE and Sch B treatment significantly reduced serum IgE levels (**Figure 5A**). Concurrently, the number of mast cells and the levels of histamine and tryptase released after degranulation were also markedly decreased in the skin of mice treated with SCSE or Sch B (**Figures 5B–E**). Moreover, serum levels of IL-4, IL-6, and TNF-α in the treated mice were significantly lower than those in the model group, with high-dose SCSE showing comparable efficacy to the positive control drug dexamethasone (**Figures 5F–H**).These collective findings demonstrate that SCSE and Sch B alleviate allergic and inflammatory responses in AD mice by suppressing IgE-mediated mast cell degranulation, thereby reducing the release of histamine, tryptase, and related inflammatory factors.

### 3.6 SCSE and Sch B suppressed NF-κB pathway activation in skin lesions of AD mice

**Figure 6:**
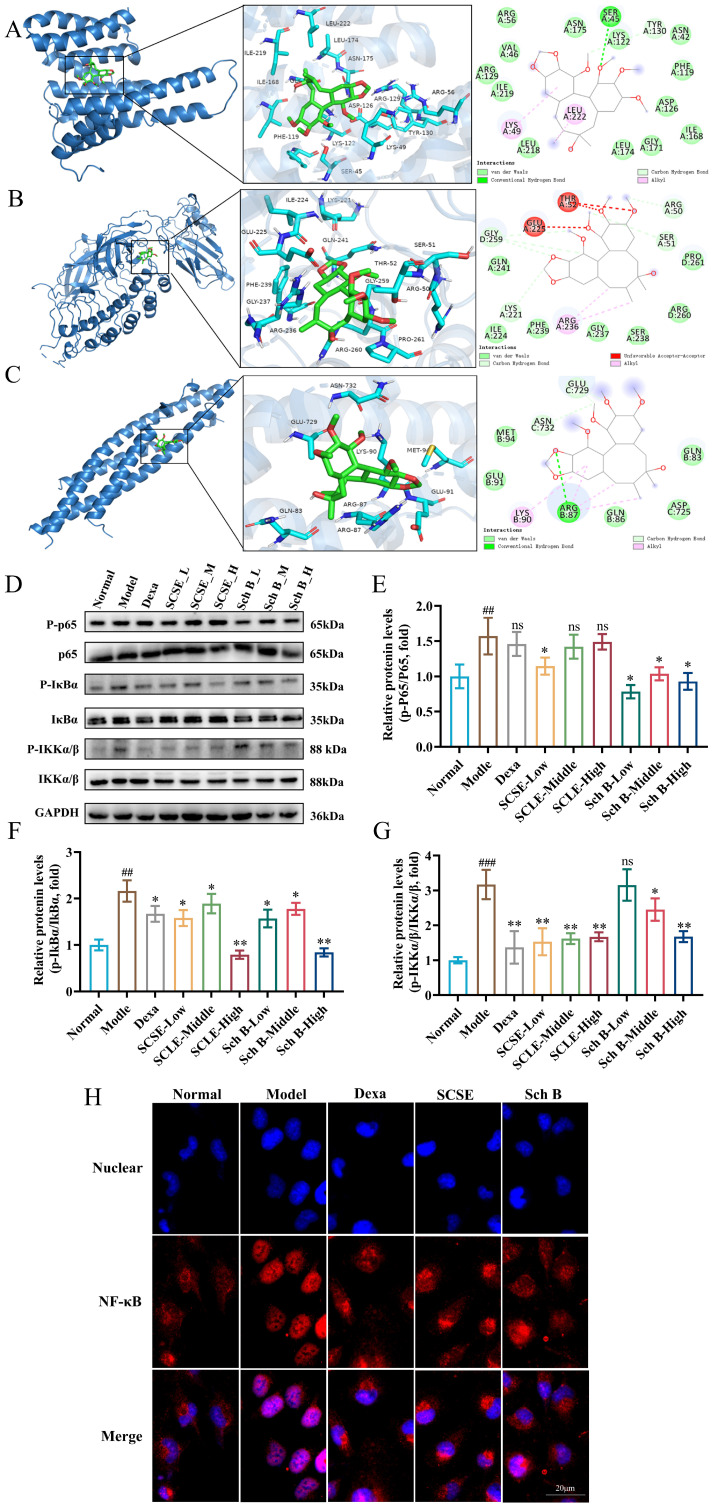
SCSE and Sch B inhibited the activation of NF-κB pathway in skin lesions of AD mice. **(A)** Molecular models of Sch B binding to the proteins of p65 **(A)**, IκBα **(B)** and IKKα/β **(C)**. The phosphorylation protein levels of p65, IκBα and IKKα/β in skin, **(D)** Western blot, **(E)** to **(G)** quantitative analysis of band intensity. Actin was used as a reference protein. **(H)** Immunofluorescence staining of DNP-IgE/DNP-BSA co-stimulated RBL-2H3 cells treated with SCSE and Sch B NF-κB activation was assessed by immunostaining for p65 intracellular localization (red) overlaid with DAPI nuclear stain (blue). Data are presented as mean ± SD (n = 6 biological replicates). Differences between the control and model groups were analyzed by an unpaired two-tailed Student’s t-test. Comparisons among the model group and treatment groups were analyzed by one−way ANOVA followed by Dunnett’s test. Significance levels are indicated as: ^#^p< 0.05, ^##^p< 0.01, ^###^p< 0.001 versus the control group; *p< 0.05, **p< 0.01, ***p< 0.001 versus the model group. ns, not significant.

The NF-κB signaling pathway, which regulates immune responses, cytokine production, and inflammation, plays a pivotal role in AD pathogenesis. To evaluate the modulatory effects of SCSE and Sch B on this pathway, we utilized both molecular docking and western blotting. Molecular docking revealed that Sch B binds stably to key components of the NF-κB pathway-IκBα, IKKα/β, and p65-with binding energies of -7.4, -5.4, and -6.7 kcal/mol, respectively. The binding interfaces were stabilized primarily by hydrophobic interactions and hydrogen bonds with specific residues: IκBα (ARG236, ARG50, SER51, GLY259, LYS221), IKKα/β (ARG87, LYS90), and p65 (SER45, LYS49, LEU222, TYR130) (**Figures 6A–C**). Western blot analysis further revealed that compared with the control group, the model group showed significantly increased phosphorylation levels of p-IKKα/β, p-IκBα, and p-p65. However, treatment with SCSE and Sch B dose-dependently reversed this effect (**Figures 6D–G**). Consistent with the molecular docking results, which indicated the strongest binding affinity between Sch B and IκBα (–7.4 kcal/mol), Western blot analysis confirmed that Sch B exhibited the most potent inhibitory effect on IκBα phosphorylation. To further validate the cellular mechanism, we performed immunofluorescence staining in RBL−2H3 cells. Stimulation with DNP−IgE/DNP−BSA markedly induced nuclear translocation of NF−κB p65, indicative of pathway activation. Pretreatment with SCSE or Sch B significantly attenuated this nuclear translocation, demonstrating direct inhibition of NF−κB signaling in mast cells (**Figures 6H**). These findings collectively indicate that the anti−AD effects of SCSE and Sch B are mediated, at least in part, through suppression of the NF−κB signaling pathway in both skin tissue and specific immune cells such as mast cells.

## 4 Discussion

This study provides the first systematic evaluation of the therapeutic effects of SCSE and its primary active constituent, Sch B, on AD in mice. Chemical profiling by UPLC-Q-Exactive-Orbitrap-MS elucidated 45 constituents, predominantly lignans. Hyaluronidase inhibition assays demonstrated significant anti-allergic activity for both SCSE and Sch B, with Sch B exhibiting efficacy comparable to that of SCSE, indicating its role as a core therapeutic component. Previous studies have reported notable antioxidant and anti-inflammatory activities of lignans derived from the fruit of *Schisandra chinensis* (26–29). Our findings further confirm that the non-medicinal part, the stem, is also rich in lignans and exhibits potent anti-allergic effects in an atopic dermatitis model, thereby offering new perspectives for the comprehensive utilization of Schisandra resources.

In therapeutic research for AD, the restoration of skin barrier function represents one of the core issues. FLG plays a critical role in the generation of natural moisturizing factors (NMFs), which are essential for maintaining stratum corneum (SC) hydration. Loss-of-function mutations in FLG lead to abnormal keratinocyte differentiation, facilitate allergen penetration, and trigger immune dysregulation, typically presenting as dry and fissured skin. Additionally, dysfunction of claudin-1 compromises SC barrier function (30), while LOR maintains the integrity of intercellular connections in the stratum corneum, thereby ensuring the barrier’s sealing function (31). Consistent with previous findings that *Schisandra chinensis* fruit extract alleviates skin inflammation and enhances barrier function in AD mice (32), our results demonstrate that SCSE and Sch B effectively restored skin barrier function, as shown by reduced TEWL and upregulated expression of barrier proteins FLG, LOR, and Claudin-1. Furthermore, our study indicates that the amelioration of AD symptoms by SCSE and Sch B is mediated through repair of DNCB-induced skin barrier damage.

Intense pruritus represents a hallmark feature of AD, in which TRPV1 has been demonstrated to play a crucial role in itch perception. Activation of TRPV1 in lesional skin leads to the release of pro-inflammatory and pruritogenic mediators, including histamine, SP, and IL-31. Histamine, released upon mast cell degranulation, has been identified as a primary pruritogen in AD. Elevated IL-31 not only directly induces pruritus but also further amplifies immune responses, establishing a persistent itch-inflammation cycle. Furthermore, SP, one of the most potent endogenous pruritogenic peptides, acts as a key mediator in AD-associated pruritus through histamine-independent mechanisms (33–38). Our results demonstrate that SCSE and Sch B significantly alleviated scratching behavior and reduced levels of pruritogenic factors such as IL-31 and SP. This anti-pruritic effect is associated with the downregulation of TRPV1, a primary molecular target, in both sensory neurons of the DRG and cutaneous nerve endings, as well as in local skin cells including keratinocytes and infiltrating immune cells. Thus, SCSE and Sch B likely mitigate pruritus by concurrently modulating TRPV1 expression across these neural and cutaneous cellular compartments. These findings further validate TRPV1 as a therapeutic target for AD. Molecular docking suggests that Sch B may bind to key residues within the intracellular hydrophobic pocket of TRPV1, which could potentially modulate channel conformation or activity. The observed downregulation of TRPV1 protein expression is correlated with the alleviation of pruritus. However, based on the docking and expression data, the interpretation that this downregulation involves direct induction of conformational destabilization and/or receptor internalization remains to be confirmed. Future experiments, such as immunofluorescence co-localization, membrane/cytosol fractionation, or surface protein biotinylation assays, would be required to test this hypothesis. If validated, this potential mechanism might alleviate pruritus while avoiding the heat sensation associated with traditional TRPV1 antagonists.

In summary, this study demonstrates the therapeutic potential of SCSE and its core constituent Sch B against AD. Both were shown to ameliorate AD-like symptoms by repairing skin barrier function, alleviating pruritus, and suppressing key inflammatory and allergic responses. The overall efficacy of SCSE likely stems from the combined actions of its multiple constituents, with Sch B playing a central role. Mechanistically, an integrated approach delineated a multi-target profile involving modulation of the TRPV1 channel and NF-κB signaling pathway.

Specifically, in terms of immunoregulation, mast cells play a pivotal role in AD by releasing inflammatory mediators. These mediators contribute to the initiation and perpetuation of inflammatory responses in the skin, leading to symptoms such as pruritus and swelling (39, 40). Our *in vivo* studies demonstrate that SCSE and Sch B effectively inhibit IgE-mediated mast cell degranulation, reduce the release of mediators such as histamine and tryptase, and markedly suppress serum levels of inflammatory cytokines including IL-4, IL-6, and TNF-α. Critically, *in vitro* experiments using the RBL-2H3 mast cell model revealed that SCSE and Sch B directly suppressed antigen-induced nuclear translocation of NF-κB p65, which is a hallmark of canonical NF-κB pathway activation, thereby identifying the NF-κB signaling within mast cells as a direct pharmacological target. Furthermore, NF-κB serves as a central pathway regulating skin inflammation, immune homeostasis, and barrier function, and its overactivation is closely associated with chronic inflammation in AD (41–43). We found that SCSE and Sch B exert anti-inflammatory effects by inhibiting NF-κB signaling pathway activation, significantly suppressing the expression and phosphorylation of p65, IKKα/β, and IκB-α in the skin of DNCB-induced AD mice, with the most potent inhibitory effect observed on IκB-α. Network pharmacology analysis further supported that the targets of Sch B encompass TRP channels and the NF-κB pathway, among other AD-related signaling pathways. From multiple perspectives, our study elucidates the molecular mechanisms by which SCSE and Sch B alleviate AD.

This study has several limitations that should be acknowledged. While we observed a significant reduction in serum IgE levels following SCSE and Sch B treatment, the upstream cellular mechanisms driving this effect, specifically the potential modulation of T helper 2 (Th2) cells, follicular helper T (Tfh) cells, and germinal center B cells in secondary lymphoid organs, were not directly investigated. Our experimental design primarily focused on elucidating the local effects within the skin. Additionally, while TRPV1 expression was downregulated, the precise molecular link between Sch B binding and this effect, including the specificity of interaction and the exact mechanism of action, remains to be established. Functional validation of TRPV1 channel inhibition, such as through calcium influx assays, was also not performed. Future studies employing techniques like flow cytometry, surface plasmon resonance (SPR), co-immunoprecipitation, TRPV1 promoter-reporter assays, or functional channel readouts will be crucial to clarify these mechanisms and further substantiate the translational potential of SCSE and Sch B for the clinical management of AD.

## 5 Conclusion

Our findings demonstrate that SCSE and its core constituent Sch B significantly ameliorated skin barrier function, reduced scratching behavior, and suppressed inflammatory responses in a murine AD model. Mechanistic studies revealed that these effects were mediated through upregulation of barrier proteins (FLG, LOR, Claudin-1), inhibition of TRPV1 channel activity, suppression of mast cell degranulation, and blockade of NF-κB pathway activation. Notably, integrated network pharmacology and molecular docking analyses identified TRPV1 and NF-κB as potential direct targets of Sch B. This study provides a scientific basis for utilizing non-traditional medicinal parts of Schisandra chinensis and proposes a promising natural product candidate for multi-target AD therapy addressing the “barrier damage-immune inflammation-pruritus” axis.

## Data Availability

The original contributions presented in the study are included in the article/**Supplementary Material**. Further inquiries can be directed to the corresponding authors.
