# Supplementary material for: The stem of Schisandra chinensis and Schisandrin B alleviated DNCB-induced atopic dermatitis in mice by inhibiting the NF-κB pathway
Source: Front Immunol. 2026 Jan 28;17:1725312. doi: 10.3389/fimmu.2026.1725312 (PMC12890654; doi:10.3389/fimmu.2026.1725312)
Supplement: Supplementary file 1 [file DataSheet1.docx]

Supplementary Material

**Supplementary Table**

Table 1 Regression data for 6 lignans analyzed by HPLC

| **compound** | **Regression equation** | **R^2^** | **Linear range（μg/mL）** |
| --- | --- | --- | --- |
| schisandrol A | y=32400x+72118 | 0.9997 | 75～600 |
| schisandrol B | y=25376x+98637 | 0.9993 | 75～600 |
| schisandrol B | y=24785x+81450 | 0.9999 | 75～600 |
| schisandrin A | y=27023x+35150 | 0.9999 | 25～300 |
| schisantherin A | y=20341x+42541 | 0.9991 | 18.5～300 |
| schisandrin C | y=23360x+2330 | 0.9999 | 18.5～300 |

**Supplementary Figures**


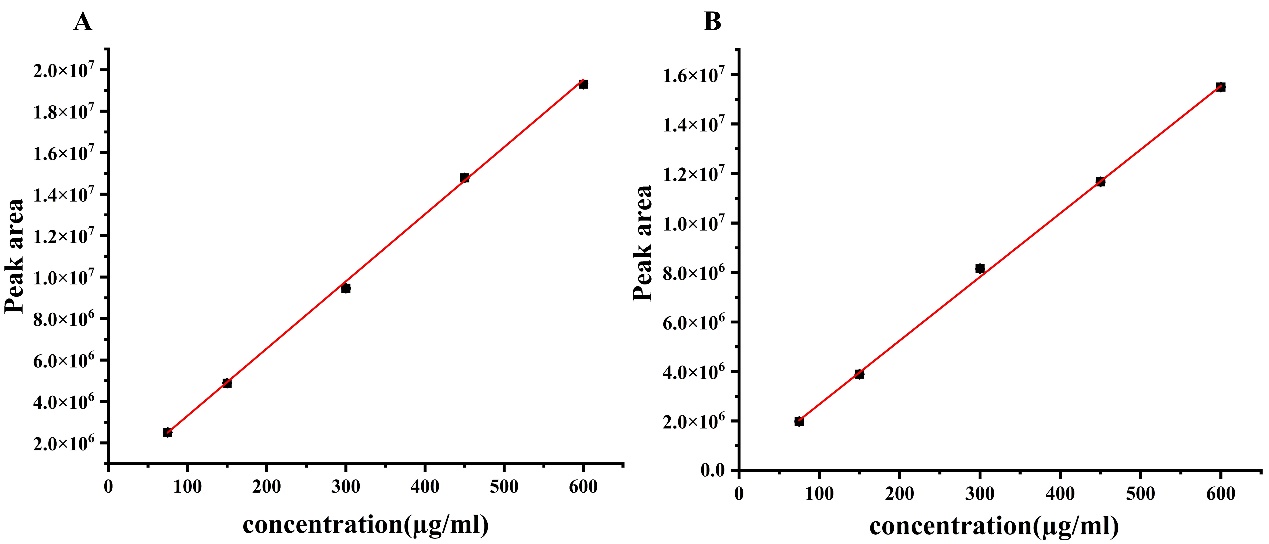

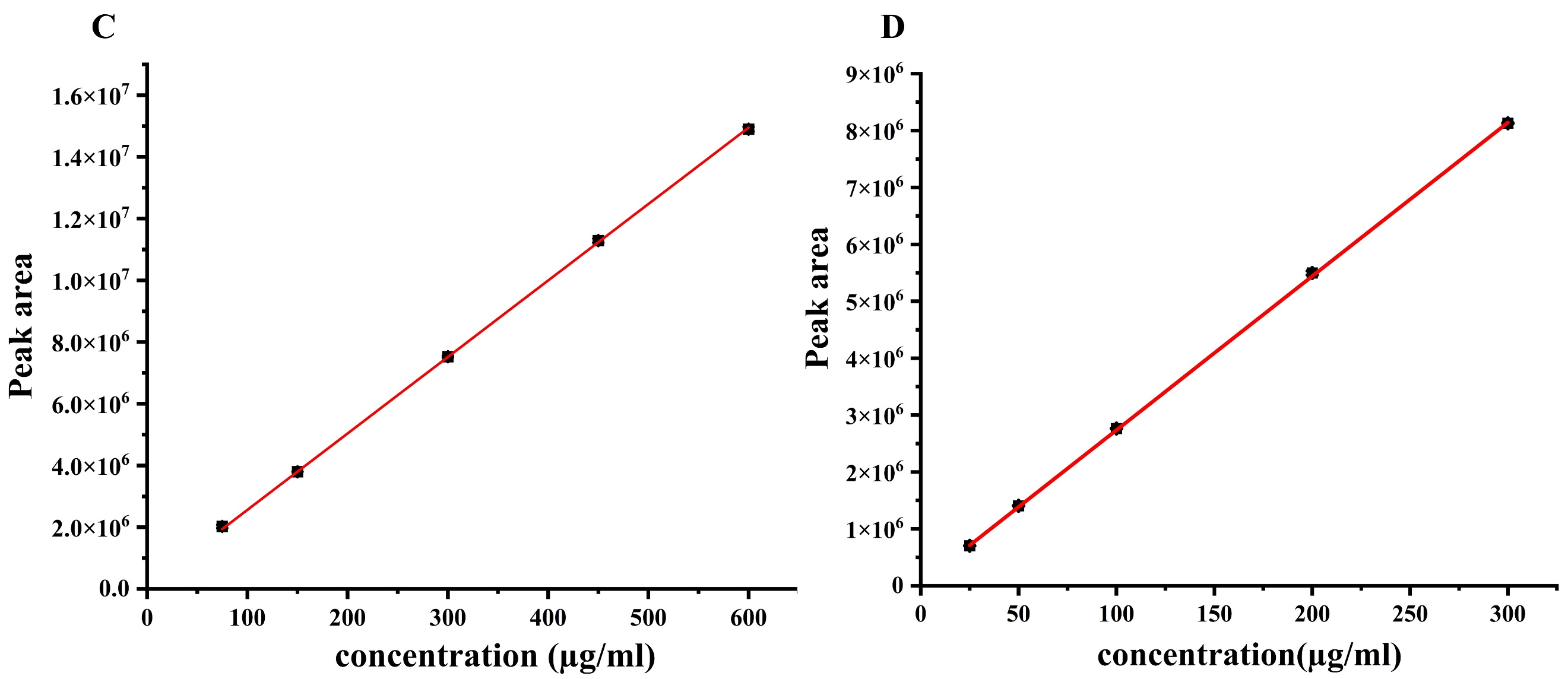

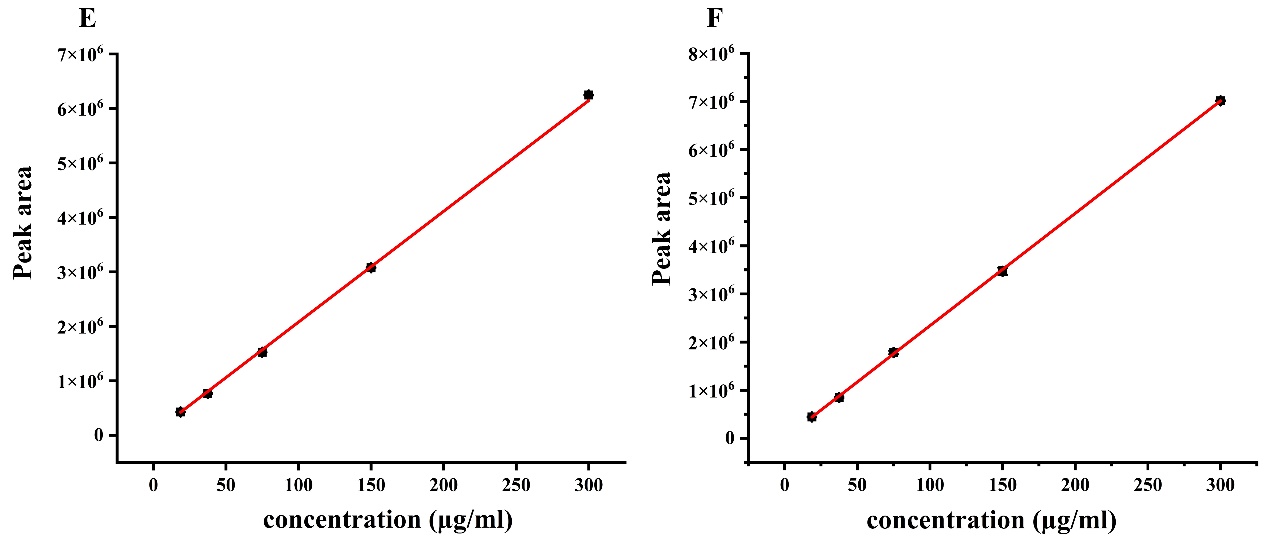


**Supplementary Figure 1.** Linear relationship curve between each standard and peak area of HPLC. Data are presented as the mean ± SD (n = 3).A. schisandrol A; B. schisandrol B; C. schisandrin B; D. schisandrin A; E. schisantherin A; F. schisandrin C.


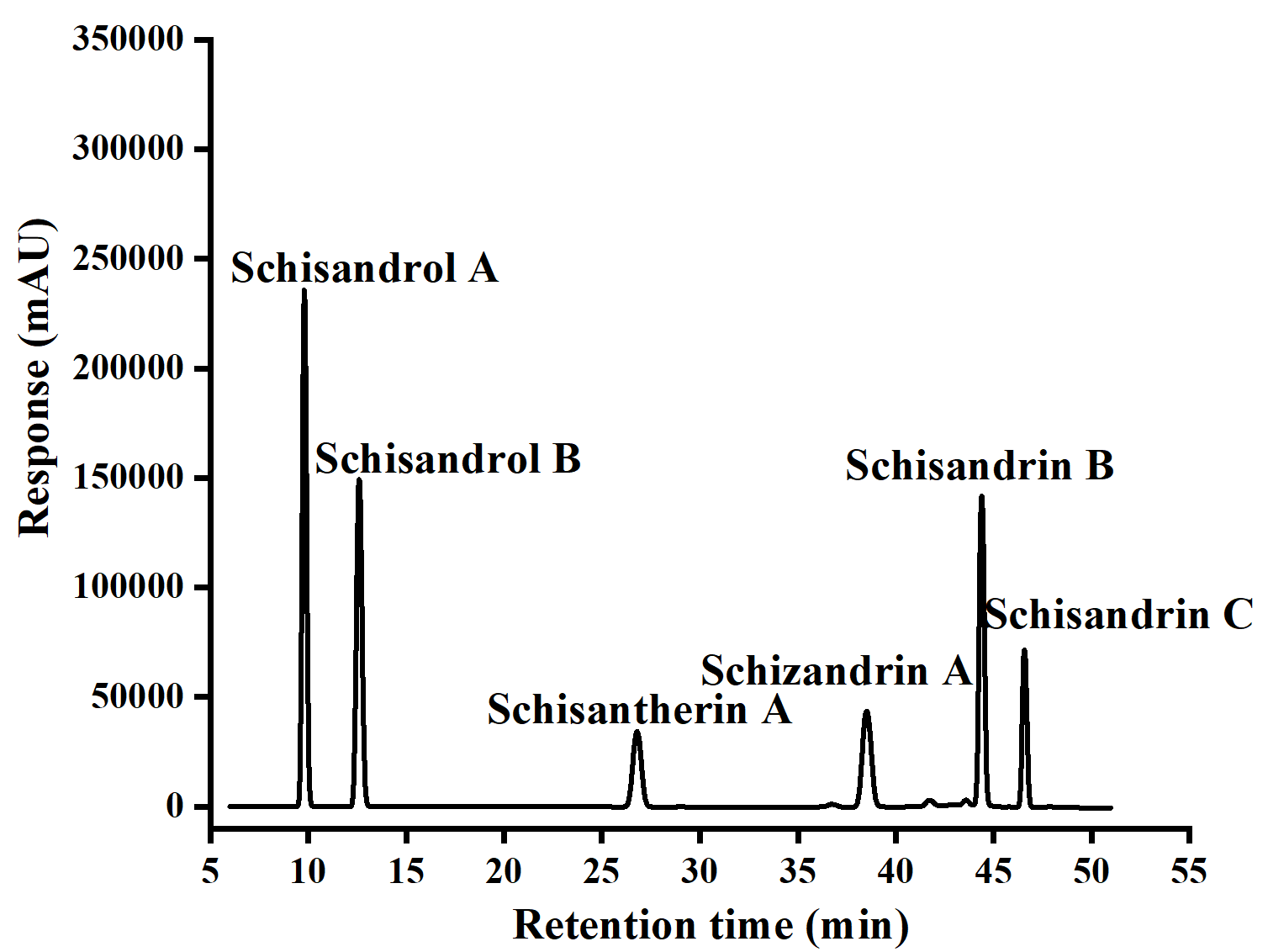


**Supplementary Figure 2.** HP LC chromatogram of mixed standard

Schisandrol A

Schisantherin A

Schizandrin A

Schisandrin B

Schisandrin C

Schisandrol B

**Supplementary Figure 3.** Chemical structures of the six major lignans identified in SCSE
